# Progression of Heart Failure in People with Type 2 Diabetes in Germany: An Analysis Using German Health Insurance Claims Data

**DOI:** 10.36469/001c.120747

**Published:** 2024-08-27

**Authors:** Keni Cheng-Siang Lee, Tobias Wagner, Adee Kennedy, Michael Wilke

**Affiliations:** 1 General Medicines Global Business Unit Sanofi, Paris, France; 2 inspiring-health GmbH, Munich, Germany; 3 Global Real World Evidence Sanofi, Bridgewater, NJ, USA; 4 Medical School Hamburg

**Keywords:** disease progression, heart failure, outcome modeling, type 2 diabetes

## Abstract

**Background:** Individuals with type 2 diabetes (T2D) show high risk of heart failure (HF). Left ventricular ejection fraction is a major factor for disease progression. In Germany, no recent longitudinal data are available. **Objectives:** To (1) measure the proportion of individuals with T2D who acquire HF over 2 years and (2) categorize ejection fraction using routine data and an algorithm, and (3) understand progression of HF in 5-year follow-up. **Methods:** This descriptive, retrospective study used longitudinal data from German statutory health insurance claims. A model using coded data classified the patients with HF into ejection fraction (EF) categories. Individuals were selected during 2013, with an inclusion period from 2014 to 2015 and a follow-up from 2016 to 2020. Baseline characteristics included demographic data, disease stage, comorbidities, and risk factors. Follow-up criteria included major adverse cardiac events (MACEs), EF category, and mortality. Disease progression was visualized by Sankey plots. **Results:** Among the 173 195 individuals with T2D identified in 2013, 6725 (median age, 74 years) developed HF in 2014 or 2015. 34.4% of individuals had MACEs, and 42.9% died over 5 years. Myocardial infarction (42%) was the most common event, followed by stroke (32%) and hospitalization (28%). A total of 5282 (78.54%) patients were classified into preserved EF and 1443 (21.46%) into reduced EF. Survival after 5 years was 71% in HF for preserved EF patients, and 29% in the HF for those with reduced EF. **Conclusion:** Heart failure is relevant in individuals with diabetes. A high number of patients may likely not survive a 5-year period. Validation of the model with German data is highly desirable. New ways of close monitoring could help improve outcomes.

## BACKGROUND

Heart failure (HF), a serious public health concern with a global prevalence of up to 40 million affected individuals, can lead to a multitude of complications and comorbidities.^1^ Further, individuals with type 2 diabetes (T2D) show a 2- to 4-fold increased risk of developing HF.^2^ Due to the increasing prevalence of T2D in Germany^3^ and a likely further increase in prevalence of HF, there is an urgent need of understanding disease progression and potential health outcomes for T2D patients suffering from HF. The treatment of HF can vary according to whether the ejection fraction (EF) is reduced or preserved (pEF),^4–6^ with a possible further subdivision into moderately reduced ejection fraction (mEF) and reduced ejection fraction (rEF).^7^ Therefore, a better understanding of the epidemiology of EF in HF progression among German patients is needed to assess the current situation in Germany. To date, no comprehensive longitudinal research has been conducted on the progression of EF in German patients with HF.

Patient records in the German healthcare system are administered largely by the statutory health insurance (SHI) companies, covering around 91% of the German population. SHI claims can serve as a decent data source for patient-based longitudinal data analyses.^8,9^ However, German SHI claims do not include measurements regarding EF. Clinical conditions are mainly available through coding of diagnosis, procedures, diagnosis-related groups (DRGs) for hospital stays, and drug prescriptions. To compensate for this lack of clinical data, we used an algorithm developed by Desai et al^10^ that was developed to predict EF class in US patients suffering from HF based on Medicare claims. The predictive variables (featuring diagnoses, procedures, and drugs) used in the algorithm can be obtained from German SHI claims. The objective of this analysis was to estimate how many individuals with T2D acquire HF over the course of 2 years and predict EF among the patients with HF during a 5-year follow-up. Another objective was to estimate disease progression (especially regarding major adverse cardiovascular events [MACEs]^11^), hospitalization, and mortality in German patients with HF over the course of 5 years.

## METHODS

### Study Design

We conducted a descriptive, retrospective study using longitudinal data from SHI claims records to show the disease progression in German individuals with T2D suffering from HF with special attention to EF class. As claims data do not contain direct clinical information, we used the predictive algorithm of Desai et al to approximate the actual EF class in German SHI patients. As a first step, the original US codes were transferred into German ICD-10 GM, OPS (operation and procedure coding for inpatient data) and ATC (anatomical therapeutic chemical coding for drugs prescribed in the inpatient and outpatient setting) codes. Then, 103 different codes were used to generate 34 predictor variables that would form the basis for the algorithm (see **Supplementary Table: Model Input**).

The population of interest were individuals with T2D, and inclusion criteria were:

- Prevalent T2D diagnosis in 2013 (secured through the minimum 2 quarters [M2Q] criteria)
- No coding of HF in 2013
- HF was first coded (as a proxy for diagnosis) in 2014 or 2015 (again using the M2Q criteria)
- We then used the years 2016-2020 for the 5-year follow-up analysis.

The algorithm allowed differentiating EF in terms of pEF, mEF, and rEF. However, due to a more efficient performance of a binominal logistic classification model (differentiation between pEF and rEF), binary classification was incorporated into this analysis. This was in line with the recommendations by Desai et al, as the mrEF group in the original study had no good correlation with the gold standard.^10^ Each predictor variable had a regression coefficient, and all these coefficients were summed. The definitions of all variables used as model inputs, together with an R programming code with all regression coefficients used per variable are provided in the **Supplementary Material**. Patients with a threshold less than 0.4678 were assigned to pEF, and those with a threshold of at least 0.4678 were assigned to rEF according to the breakpoints used by the authors who developed the algorithm. The primary outcomes during the follow-up period included the number of patients with HF with preserved EF and reduced EF (HFpEF and HFrEF, respectively) during an observation period of 5 years. The secondary outcomes were the number of HFpEF/HFrEF patients developing MACEs (excluding cardiovascular [CV] death), number of hospitalizations, CV death (coded), and death for other (coded) reasons. Sankey plots were used to visualize disease progression and outcomes during the follow-up. These plots allow to visualize the “flow” of patients from one group to another over time and provide insights into several patient pathways and transition from one state to another in a single visualization.^12^

### Data Source

We selected patient claims data provided by the Deutsche Analysedatenbank für Evaluation und Versorgungsforschung (German analytical database for evaluation and healthcare research; DADB) provided by the Gesundheitsforen Leipzig (GFL).^13^ DADB is a comprehensive database containing longitudinal data of approximately 3.5 million German individuals insured in the German SHI system from 2013 to 2020. SHI claims contain patient-based data regarding ICD codes, OPS codes, EBM codes (operation and procedure coding for outpatient data), and ATC codes, allowing to follow patient pathways and disease progression.

### Extrapolation

The data source allowed an extrapolation on the total SHI population (≈91% of the German population) using standardized age and sex tables. These tables are released by the statistics office and used broadly for extrapolation. Instead of using only one multiplier factor, the factors were adjusted based on the age and sex distribution of the study population compared with the total German population. If, for example, the data set comes from AOK insurance, patients are often older and comprise more women than the data set from BMW insurance. Using the individual insurance-weighted factors therefore allowed a more exact extrapolation. After the analysis, we performed the extrapolation to state the potential burden of disease on the German SHI population.

## RESULTS

### Study Population

**Figure 1.**
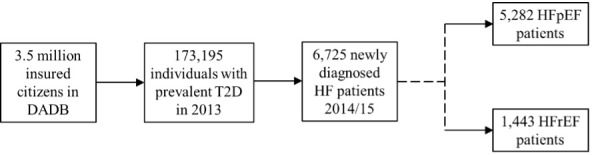
Flowchart of Patient Inclusion and Classification Abbreviations: DADB, Deutsche Analysedatenbank für Evaluation und Versorgungsforschung; HF, heart failure; HFpEF, heart failure with preserved ejection fraction; HFrEF, heart failure with reduced ejection fraction; T2D, type 2 diabetes.

A total of 173 195 individuals meeting the inclusion criteria were selected and followed up to observe whether they would develop HF during a 2-year inclusion period. The final study population derived from the explorative analysis consisted of a comprehensive data set, including data for 6725 patients with HF, who were followed up over a 5-year period from 2016 to 2020 (**Figure 1**), indicating that approximately 4% of the individuals with T2D may develop HF in this 2-year period. In the first year (the index year), 5282 patients were classified as HFpEF patients (78.5%) and 1443 as HFrEF patients (21.5%).

The baseline characteristics in our study included demographic data, disease stage, comorbidities, and relevant risk factors. In our analysis, individuals with T2D were on median 8 years younger than their HF counterparts (66 years vs 74 years), and sex was distributed similarly (**Table 1**). Patients with HF showed an increased percentage of having CV and metabolic risk factors, indicating an overall worsened state of health. Overall, 12% of the individuals in the T2D population and 6% of the patients with HF did not feature any relevant comorbidities at the onset of the follow-up period. Approximately 50% of the patients with HF had metabolic and CV risk factors. This comorbidity complex was also represented in the Charlson Comorbidity Index (CCI).^14^ The prevalent T2D population had a median CCI of 2 vs 5 in those with HF. Further, patients with HF show an increased likelihood of being prescribed sodium-glucose transport protein 2 (SGLT-2) inhibitors.

**Table 1.** Baseline Characteristics

**Variable**	**Prevalent T2D Population (2013)**		**HF Population (2014/2015)**	
	**n**	**%**	**n**	**%**
Total No. of patients	173 195	-	6725	3.88
Age (median, IQR), y	66 (8, 5)		74 (7, 5)	
Sex (male/female)	100 039/73 156	57.76/42.24	4062/2663	60.40/39.60
Charlson Comorbidity Index (median, IQR)	2 (1, 5)		5 (1, 5)	
Metabolic risk factors				
Obesity	49 212	28.41	2220	33.01
Any hyperlipidemia	60 927	35.18	2639	39.24
CV risk factors				
Hypertension	136 510	78.82	5893	87.63
Myocardial infarction	5197	3.00	396	5.89
Chronic ischemic heart disease	43 240	24.97	3017	44.86
Cerebrovascular	11 752	6.79	685	10.19
T2D with/without comorbidity	21 317	12.31	412	6.13
T2D with metabolic risk factors	10 822	6.25	161	2.39
T2D with CV risk factors	68 108	39.32	2868	42.65
T2D with CV + metabolic risk factors	72 948	42.12	3284	48.83
SGLT2 inhibitor prescription	323	0.19	85	1.26
NYHA_1 (n, % of total HF)	–	–	345	5.13
NYHA_2 (n, % of total HF)	–	–	895	13.31
NYHA_3 (n, % of total HF)	–	–	1170	17.40
NYHA_4 (n, % of total HF)	–	–	916	13.62
NYHA_not_coded (n, % of total HF)	–	–	3399	50.54
HFpEF (n, % of total HF)	–	–	5282	78.54
HFrEF (n, % of total HF)	–	–	1443	21.46

Abbreviations: CV, cardiovascular; HF, heart failure; HFpEF, heart failure with preserved ejection fraction; HFrEF, heart failure with reduced ejection fraction; IQR, interquartile range; NYHA, New York Heart Association; SGLT2, sodium-glucose transport protein 2; T2D, type 2 diabetes.

### Predictor Variables for the Algorithm

The predictor variables classifying EF were divided into 4 basic categories: demographic variables, prescribed medication, comorbidities, and HF-related variables (**Table 2**). Comorbidities formed the most comprehensive category with a total of 19 variables, followed by prescribed drugs (7 variables), HF-related variables (4 variables) and demographic variables (4 variables). The most frequent comorbidity was hypertension, affecting 95% of the patients with HF, followed by hyperlipidemia (64%) and stable angina pectoris (42%). The most frequently coded drugs were β-blockers (72% of the total HF population), loop diuretics (58% of the total HF population), and ACE inhibitors (46% of the total HF population). The most frequent HF-related variable was left HF (56%). Most predictive factors were more frequent in the HFrEF group, which is coherent with the logic of a predictive algorithm. However, it is worth noting that obesity, depression, systolic HF, and unspecified HF were less prevalent in the HFrEF group than in the HFpEF group.

**Table 2.** Distribution of Predictor Variables After Applying the Algorithm

**Variables**	**LVEF Category Assigned (by Algorithm)**			
	**HFpEF, n/Mean**	**HFpEF (%)**	**HFrEF, n/Mean**	**HFrEF (%)**
No. of patients	5282	78.54	1443	21.46
male	2973	56.29	1089	75.47
index_dx_out	3863	73.14	250	17.33
age1	73.31	–	71.68	–
dx_defibrillator	62	1.17	104	7.21
hosp_chf1	0.28	–	0.84	–
rx_ace	2142	40.55	924	64.03
rx_antagonist	534	10.11	449	31.12
rx_bblocker	3632	68.76	1198	83.02
rx_digoxin	6	0.11	3	0.21
rx_loop_diuretic	2784	52.71	1120	77.62
rx_nitrates	247	4.68	93	6.44
rx_thiazide	601	11.38	152	10.53
dx_afib	1650	31.24	697	48.30
dx_anemia	549	10.39	143	9.91
dx_cabg	70	1.33	79	5.47
dx_cardiomyopathy	146	2.76	420	29.11
dx_copd	1307	24.74	396	27.44
dx_depression	1315	24.90	294	20.37
dx_htn_nephropathy	364	6.89	130	9.01
dx_hyperlipidemia	3298	62.44	997	69.09
dx_hypertension	5019	95.02	1387	96.12
dx_hypotension	159	3.01	65	4.50
dx_mi	255	4.83	436	30.21
dx_obesity	2230	42.22	576	39.92
dx_oth_dysrhythmia	1055	19.97	437	30.28
dx_psychosis	66	1.25	13	0.90
dx_rheumatic_heart	445	8.42	149	10.33
dx_sleep_apnea	552	10.45	161	11.16
dx_stable_angina	2866	54.26	1061	73.53
dx_valve_disorder	60	1.14	37	2.56
hf_systolic	638	12.08	732	50.73
hf_diastolic (no ICD in Germany)	–	–	–	–
hf_left	2450	46.38	1347	93.35
hf_unspecified	2414	45.70	31	2.15
median pv score	0.24	–	1.55	–
mean pv score	0.23	–	1.45	–

Abbreviations: CABF, coronary artery bypass surgery; CHF, chronic heart failure; dx, diagnosis; COPD, chronic obstructive pulmonary disease; HF, heat failure; HFpEF, heart failure with preserved ejection fraction; HFrEF, heart failure with reduced ejection fraction; ICD, International Classification of Diseases; LVEF, left ventricular ejection fraction; Rx, prescription.

### Disease Progression

**Figure 2.**
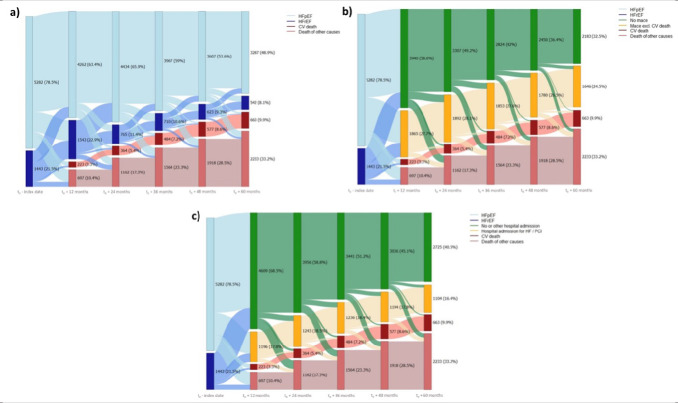
Sankey Plots: (**a**) Distribution of HFpEF/HFrEF in the Follow-up; (**b**) Incidence of MACE; (**c**) Hospitalization Abbreviations: CV, cardiovascular; HFpEF, heart failure with preserved ejection fraction; HFrEF, heart failure with reduced ejection fraction; MACE, major adverse cardiovascular events.

One major objective of our analysis was determining the disease progression of EF in patients with HF. Over 5 years, the proportion of patients having HFpEF decreased from 79% to 49% (**Figure 2a**), whereas the proportion of patients having HFrEF decreased from 22% to 8%. This was mainly due to an overall 5-year mortality rate of 43%. A CV ICD-10 was coded as the cause of death for 10% of the patients, while other ICDs were coded for 33% of the patients. According to our analysis, approximately half of the individuals with T2D and HF did not survive a 5-year follow-up period. Further, our analysis shows a progression of HFpEF patients to HFrEF and vice versa. Overall comorbidities can change in a patient pathway, and our analysis indicates that patients’ conditions improve and worsen over the course of 5 years. Further, it appears that HFrEF patients do not remain stable for a longer period; either their EF improves to a pEF, or they are likely to die. Among the surviving patients with HF, 86% (3287/3829) were classified as HFpEF patients and 14% (542/3829) as HFrEF patients.

Approximately 33% of the patients with HF manage to survive 5 years without having a MACE (**Figure 2b**). After 5 years of follow-up, approximately 25% of the patients with HF had a MACE excluding death, and 10% died of CV events, resulting in 35% of the patients with HF experiencing a MACE. Of interest, after 12 months, only about 59% of the patients with HF did not have a MACE or died. Also, a sizeable portion of HFpEF patients had experienced MACE already after 12 months. The most common MACE was myocardial infarction (42%), followed by stroke (32%), hospitalization for HF (28%), and death due to CV causes.

About 41% of patients with HF were not hospitalized over the course of 5 years, dropping from about 69% after 1 year of follow-up (**Figure 2c**). The rate of hospitalized patients remained stable over the follow-up period, ranging between 16% and 18%. Similar to the MACE plot, a sizeable proportion of HFpEF patients underwent an HF-related hospitalization already after the first year of follow-up.

Disease progression in patients with HF is associated with a high mortality rate of approximately 43% over 5 years of follow-up and a high likelihood of MACEs. Further, HFpEF patients showed more favorable overall disease progression than HFrEF patients.

The 6725 individuals with T2D newly diagnosed with HF in the DADB can be extrapolated to 263 361 patients in the German SHI population. The mortality translates into 27 979 CV deaths and 95 104 deaths with other recorded causes. Within a 5-year time period, 138 260 individuals with T2D would have suffered from MACE and 90 540 patients would have been admitted to hospital for HF. Extrapolating our findings on the hospitalizations to the German SHI population translates into 210 674 newly diagnosed pEF patients and 52 686 rEF patients with 123 083 patients dying within 5 years of the diagnosis and 172 821 patients with at least 1 hospital stay.

## DISCUSSION

The incorporation of the algorithm by Desai et al into a German data set showed promising results. We compared the distribution of predictive variables in our study population with the US validation cohort of Desai et al. The overall distribution of the EF classes seemed to be fairly similar to the US population with a known EF classification (≈76% of the patients with HF being classified as HFpEF and ≈24% being classified as HFrEF in the US data vs ≈79% HFpEF and ≈21% in the German algorithm-based classification) (**Table 3**). The sample size used to validate the Desai algorithm was comparable. The US population with a known left ventricular ejection fraction (LVEF) class included 7001 patients, and the German population comprised 6725 patients. However, visible differences were observed in the distribution of some predictive variables, which may be attributed to different coding preferences (left HF and stable angina being much more frequently coded in the German data while valve disorders and other dysrhythmias being more frequent in the US data), different drug prescription routines (β-blockers being more commonly prescribed in Germany vs thiazide diuretics being more frequently prescribed in the United States) and differences in lifestyle-related health outcomes in the respective samples (obesity and psychosis). Despite the differences in the distribution of predictive variables, the overall EF classification indicates a high predictive power of the Desai et al algorithm in the German data.

**Table 3.** Comparison of German Algorithm–Classified EF with US Measured EF

	**Percentage**				**Absolute Numbers**			
	**Percentage HFpEF Assignment**		**Percentage HFrEF Assignment**		**HFpEF Assigned (n)**		**HFrEF Assigned (n)**	
**Variable**	**US Gold Standard**	**German Algorithm**	**US Gold Standard**	**German Algorithm**	**US Gold Standard**	**German Algorithm**	**US Gold Standard**	**German Algorithm**
HFpEF/HFrEF in the study population	75.72	78.54	24.28	21.46	5301	5282	1700	1443
Demographics								
Male	50.69	56.29	67.76	75.47	2687	2973	1152	1089
Age (y), mean	70.6	73.3	69.2	71.7	70.6	73.3	69.2	71.7
HF-specific ICD-10 codes								
Systolic HF	8.98	12.08	38.65	50.73	476	638	657	732
Diastolic HF	25.66	–	4.88	–	1360	–	83	–
Left HF	4.51	46.38	5.53	93.35	239	2450	94	1347
Unspecified HF	55.27	45.70	46.47	2.15	2930	2414	790	31
Implantable cardioverter- defibrillator	2.09	1.17	14.41	7.21	111	62	245	104
HF diagnosis identified in outpatient claims	59.35	69.73	52.12	17.33	3146	3683	886	250
HF-related medication								
ACE inhibitors	41.49	40.55	56.94	64.03	2108	2142	968	924
Mineralocorticoid receptor antagonists	10.18	10.11	22.88	31.12	467	534	389	449
β-blockers	49.61	68.76	58.71	83.02	2587	3632	998	1198
Digoxin	2.82	0.11	5.94	0.21	118	6	101	3
Loop diuretics	48.62	52.71	56.00	77.62	2489	2784	952	1120
Nitrates	10.40	4.68	16.76	6.44	519	247	285	93
Thiazide diuretics	30.65	11.38	37.00	10.53	1581	601	629	152
Comorbidities								
Atrial fibrillation or flutter	36.90	31.24	42.53	48.30	1956	1650	723	697
Anemia	40.01	10.39	34.29	9.91	2121	549	583	143
Coronary artery bypass graft	5.51	1.33	7.76	5.47	292	70	132	79
Cardiomyopathy	10.79	2.76	46.41	29.11	572	146	789	420
COPD	29.03	24.74	24.82	27.44	1539	1307	422	396
Depression	17.75	24.90	12.29	20.37	941	1315	209	294
Hypertensive nephropathy	14.56	6.89	14.18	9.01	772	364	241	130
Hyperlipidemia	63.31	62.44	62.53	69.09	3356	3298	1063	997
Hypertension	82.53	95.02	80.29	96.12	4375	5019	1365	1387
Hypotension	15.30	3.01	17.24	4.50	811	159	293	65
Myocardial infarction	11.47	4.83	25.65	30.21	608	255	436	436
Obesity	24.09	42.22	19.06	39.92	1277	2230	324	576
Other dysrhythmias	46.58	19.97	58.94	30.28	2469	1055	1002	437
Psychosis	37.05	1.25	31.71	0.90	1964	66	539	13
Rheumatic heart disease	18.75	8.42	15.29	10.33	994	445	260	149
Sleep apnea	17.92	10.45	13.82	11.16	950	552	235	161
Stable angina	10.19	54.26	12.65	73.53	540	2866	215	1061
Valve disorders	21.66	1.14	16.35	2.56	1148	60	278	37

Abbreviations: CABF, coronary artery bypass surgery; CHF, chronic heart failure; dx, diagnosis; COPD, chronic obstructive pulmonary disease; HF, heart failure; HfpEF, heart failure with preserved ejection fraction; HfrEF, heart failure with reduced ejection fraction; ICD, *International Classification of Diseases*; LVEF, left ventricular ejection fraction; Rx, prescription.

Our findings are in line with other publications on the distribution of HFrEF and HFpEF. It is well known that HFrEF is more prevalent in men than in women.^15^ In our cohort, 75% of all prevalent HFrEF patients were men, while in the HFpEF cohort, men accounted for about 56% of the patients. Also, the age distribution of HFpEF/HFrEF aligns with established findings in the literature.^16^

Individuals with T2D undergo a rapid progression of HF. As shown in **Figure 2**, only approximately 50% of patients still showed a pEF according to the algorithm. Mortality increased each year, while CV-related deaths remained at around 23% of all deaths, which could be an indication of a high level of multimorbidity in T2D patients. This is to be expected, especially given the increased age of the patients in our study, and aligns with findings in the current literature.^4^ The mortality rate of approximately 13% after only 12 months (with a sizeable proportion of deaths being attributed to the pEF classification) might be due to the initial diagnosis of HF being assigned to patients’ records while HF was already present, before the onset of the follow-up, and thus resulting in an already worsened condition of the patients.

**Figure 3.**
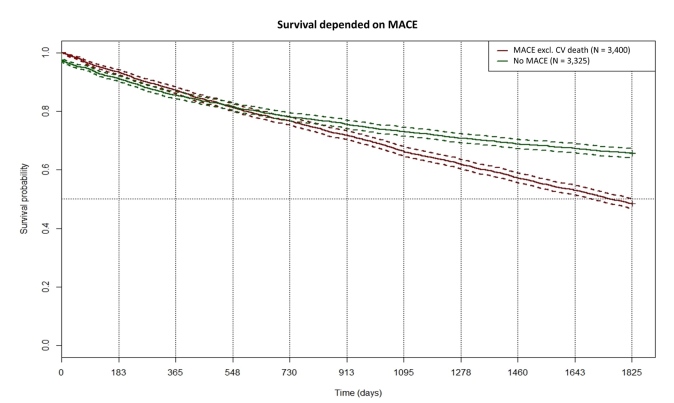
Kaplan-Meier Curve: Survival Probability vs Occurrence of MACE Abbreviations: CV, cardiovascular; MACE, major adverse cardiovascular events.

MACEs (excluding CV death) occurred in nearly 50% of HFrEF patients and 25% of HFpEF patients within the first year. Since the definition of MACE usually includes death from CV disease, we continued to use the differentiation of death caused and not caused by CV diseases and therefore split the MACE category. CV-related death occurred in 3.3% of the total population. Data from a Chinese registry report lower rates with 12.9% for HFrEF and 2.9% for HFpEF.^17^ However, this registry saw higher rates of CV death than those presented in our study. Dai et al reported a 6-month MACE rate of 22.09%, while in our data, a total of 35% of patients with HF experience MACEs within 12 months.^18^ The higher rates in our population can either be due to the fact that we focused on T2D patients or that the coding of HF in the health insurance database occurred when the disease was already prevalent for some time. We computed a Kaplan-Meier curve for the survival probability dependent on MACE (excluding CV death; **Figure 3**). In the first 2 years, the occurrence of MACEs did not influence survival probability. On the 5-year trajectory, patients with MACE have a significantly lower survival probability (48.5% vs 72.3%; *P* < .0001, log-rank test). The occurrence rate of MACEs in HF is knowingly driven by further confounding factors such as medication adherence, socioeconomic status, or medication adherence. As the data set from the health insurance did not contain information on these factors, we could not calculate their impact, which is a limitation of this study.

Hospitalization was frequent among T2D patients with HF. In our analysis, it was only possible for hospitalized patients to move to the deceased category to retain a category of never-hospitalized patients. Thus, the number of patients being hospitalized each year for the first time would equal the number of patients dying in the same period, creating a stable influx and efflux. More than 60% of all patients had at least 1 CV-related hospital stay during the 5-year follow-up period. This is in line with other findings regarding the progression of HF and hospital stays.^1,19^

Over years, the Desai et al algorithm has been adopted by various research groups and has confirmed its validity.^20–22^ However, some limitations are related to the Desai algorithm. This algorithm is based on coded ICD-10 and medication data. If certain patient comorbidities or conditions are not coded or certain drugs are not prescribed, the algorithm might assign a patient to the wrong group. In the original publication, the authors found a correct assignment in 83% of the cases,^10^ indicating a certain number of wrongly assigned cases. The distribution in frequency of the examined variables as outlined in **Table 3** shows some variations in the US gold standard (based on clinical data), which might also skew the results. The use of a predictive algorithm is only an approximation of the clinical EF classification, leaving room for coincidence and bias. Thus, a validation of the algorithm-based EF classification with measured EF of German patients would be highly desirable.

## CONCLUSION

Heart failure in individuals with T2D leads to a substantial burden of disease in Germany. Based on the analysis, about 263 000 (3.9%) individuals acquire HF over 2 years; of these individuals, over 120 000 (43%) may likely die in the following 5 years after HF diagnosis. Many patients have at least 1 hospital visit. The LVEF categorization using routine data should be validated with German clinical data. Closer digital monitoring of these patients might help to slow down the progression of HF.

### Disclosures

K.C.-S.L. and A.K. are employees of Sanofi and may hold shares and/or stock options in the company. T.W. does not have any conflicts of interest. M.H.W. is CEO of inspiring-health, which received funding for this research from Sanofi; has received grants from Abbott for health economic study and from Fractyl for PMCH-Study; consulting fees for health economic analyses from Abbott, Fractyl, gbo Medizintechnik, and Biocomposites; honoraria from Novartis for lectures, Advisory Board participation for UNEEG. M.H.W. is also peaker of the Economic Aspects of Anti-infective Therapies working group for Paul-Ehrlich Gesellschaft e.V. and holds personal stocks in Pfizer, Johnson & Johnson, and BioNtech.

## Supplementary Material

Supplemental Tables Table S1: Model input
Table S2: Patient characteristics
Table S3: Outcomes progression
